# Supplementary material for: Simulating metagenomic stable isotope probing datasets with MetaSIPSim
Source: BMC Bioinformatics. 2020 Jan 30;21:37. doi: 10.1186/s12859-020-3372-6 (PMC6993524; doi:10.1186/s12859-020-3372-6)
Supplement: Supplementary file 1 — Additional file 1: Supplementary Materials and Methods. Table S1. MetaSIPSim dependencies. Table S2. Parameters used for the MetaSIPSim validation and case study simulations. * Values may differ between simulations as described in the materials and methods. Table S3. Processing times for all simulations from the cases study. All times are in seconds. Table S4. Summary statistics for recovery of reads, contigs, and MAGs for labeled genomes in initial simulations. Figure S1. Visual description of variables defined in the manuscript and Table 2. Thick blue line indicates the position of a single DNA fragment during and after ultracentrifugation. Centrifugation setup shown here is based on a fixed angled rotor. Figure S2. Diagram of experimental design for case study simulations. Figure S3. RefSeq genomes G + C distribution with G + C bins rounded to nearest whole number (downloaded January 25, 2019). Figure S4. G + C distributions for the 500 genomes in each reference set. G + C bins are rounded to the nearest whole number. Vertical line indicates G + C skew of the set. Figure S5. Rank-abundance plots for each simulated sample or replicate community including the 12C-control (Con) and all five 13C-trials (T1-T5). Random genomes are only labeled in the 13C-labeled samples. Vertical grey lines indicate ranks of the 50 labeled genomes per treatment. Labeled genomes were selected such that mean ranks of the labeled genomes averaged across all samples were similar across the reference sets (lowGC, medGC, and highGC). Figure S6. Fold difference in raw read coverage for each labeled genome between the metagenomic-SIP and shotgun metagenomic libraries from the original simulations. Values above one indicate greater coverage in the metagenomic-SIP compared to the shotgun metagenomic libraries. Figure S7. Fold difference in proportion of each labeled genome recovered by reads between the metagenomic-SIP and shotgun metagenomic libraries from the original simulations. Values abov [file 12859_2020_3372_MOESM1_ESM.docx]

**Simulating metagenomic stable isotope probing datasets with MetaSIPSim**

Samuel E. Barnett^1*^ and Daniel H. Buckley^1^

**Supplementary Materials and Methods**

MetaSIPSim is designed to simulate next generation sequencing reads from Illumina technologies. However, other technologies, such as long read sequencers from PacBio and Oxford Nanopore Technologies, can also be used for metagenomic-SIP. MetaSIPSim when combined with other read simulation tools such as those reviewed in Escalona et. al 2016 [1] or newer simulators for Oxford Nanopore [2] can be used to simulate metagenomic-SIP datasets from other sequencing technologies. To do this, MetaSIPSim should be used to generate the fragment list. The fragment list is always generated with any MetaSIPSim run so simulations can be performed for both Illumina and alternative long read sequencers at the same time. Alternatively, for rapid processing, the later steps of Illumina read generation can be skipped as they are not needed. The fragment list contains reference genome identity, start position, length, and abundance values for all fragments simulated. Using the start positions and length values, custom code implementing the python module pyfasta can generate sequence files in FASTA format for all fragments. These fragment sequences and their abundance values can then be used directly as input for many read simulation tools. The fragments would become the reference sequences and their abundance values determine the abundances of each reference fragment in the sequenced BD window or gradient fraction.

1. Escalona M, Rocha S, Posada D. A comparison of tools for the simulation of genomic next-generation sequencing data. Nat Rev Genet 2016;17, 459–469. doi:10.1038/nrg.2016.57

2. Li Y, Han R, Bi C, Li M, Wang S, Gao X. DeepSimulator: a deep simulator for Nanopore sequencing. Bioinformatics 2018;34(17):2899–2908. doi:10.1093/bioinformatics/bty223

**Supplementary Tables**

**Table S1:** MetaSIPSim dependencies

| **Python module/dependency** | **version** |
| --- | --- |
| numpy | ≥ 1.16.3 |
| pandas | ≥ 0.24.2 |
| Biopython | ≥ 1.73 |
| scipy | ≥ 1.2.1 |
| pyfasta | ≥ 0.5.2 |
| InSilicoSeq (recommended) | ≥ 1.3.6 |

**Table S2:** Parameters used for the MetaSIPSim validation and case study simulations. * Values may differ between simulations as described in the materials and methods.

| **Parameter** | **Lueders**  **et al.**  **2004** | **Buckley**  **et al.**  **2007** | **Wawrik**  **et al.**  **2009** | **Case study**  **simulations** |
| --- | --- | --- | --- | --- |
| BD window or fractions | fraction | | | window |
| Simulation endpoint | fragment list | | | read sequences |
| Sequencing window  min BD (g/ml) | NA | | | 1.72* |
| Sequencing window  max BD (g/ml) |  |  |  | 1.77* |
| Genome coverage  with fragments | 100X | | | |
| Fragment length  distribution | Skewed normal (mean = 9000, scale = 2500, shape = -5) | | | |
| Temperature (K) | 293.15 | | | |
| Average gradient  density (g/ml) | 1.725 | 1.69 | 1.701 | 1.69 |
| (angular velocity)^2^ (rad/s)^2^ | 20465612 | 33172837 | 16860743 | 33172837 |
| Min radius of tube  from axis of rotation (cm) | 7.21 | 2.6 | 7.47 | 2.6 |
| Max radius of tube  from axis of rotation (cm) | 8.49 | 4.85 | 8.79 | 4.85 |
| Angle of tube (degrees) | 0 | 28.6 | 0 | 28.6 |
| Tube radius (cm) | 0.65 | 0.66 | 0.66 | 0.66 |
| Tube height (cm) | 6.6 | 4.7 | 4.7 | 4.7 |
| Proportion of DNA  in DBL | 0.001 | | | |
| Stable isotope element | C | N | N | C |
| Min BD for model  gradient (g/ml) | 1.67 | | | 1.67* |
| Max BD for model  gradient (g/ml) | 1.775 | | | 1.775* |
| BD steps for model  gradient (g/ml) | 0.0001 | | | 0.0001 |
| Max read length (bp) | NA | | | 151 |
| Average insert size (bp) |  |  |  | 1000 |
| Standard deviation  of insert size (bp) |  |  |  | 5 |
| Final number of reads |  |  |  | 5,000,000* |

**Table S3:** Processing times for all simulations from the cases study. All times are in seconds.

| **Metagenome**  **type** | **Comm.**  **G+C** | **Seq.**  **depth** | **Library** | **Frag.^a^** | **Frag.**  **abund.^b^** | **Reads^c^** | **Library**  **total** | **Sim.**  **total** |
| --- | --- | --- | --- | --- | --- | --- | --- | --- |
| SIP | Low | 5MM | 1 | 557 | 568 | 250 | 818 | 5503 |
|  |  |  | 2 |  | 567 | 256 | 823 |  |
|  |  |  | 3 |  | 566 | 257 | 823 |  |
|  |  |  | 4 |  | 568 | 266 | 834 |  |
|  |  |  | 5 |  | 568 | 255 | 823 |  |
|  |  |  | 6 |  | 578 | 244 | 819 |  |
| shotgun |  |  | 1 | 559 | 71 | 196 | 267 | 2186 |
|  |  |  | 2 |  | 71 | 196 | 267 |  |
|  |  |  | 3 |  | 70 | 194 | 264 |  |
|  |  |  | 4 |  | 69 | 210 | 279 |  |
|  |  |  | 5 |  | 70 | 214 | 283 |  |
|  |  |  | 6 |  | 70 | 197 | 267 |  |
| SIP | Medium |  | 1 | 613 | 640 | 298 | 938 | 6008 |
|  |  |  | 2 |  | 633 | 257 | 890 |  |
|  |  |  | 3 |  | 637 | 243 | 881 |  |
|  |  |  | 4 |  | 630 | 286 | 916 |  |
|  |  |  | 5 |  | 630 | 240 | 870 |  |
|  |  |  | 6 |  | 628 | 268 | 896 |  |
| shotgun |  |  | 1 | 629 | 78 | 218 | 296 | 2331 |
|  |  |  | 2 |  | 77 | 206 | 283 |  |
|  |  |  | 3 |  | 77 | 199 | 275 |  |
|  |  |  | 4 |  | 78 | 207 | 285 |  |
|  |  |  | 5 |  | 77 | 206 | 283 |  |
|  |  |  | 6 |  | 78 | 202 | 280 |  |
| SIP | High |  | 1 | 664 | 671 | 234 | 904 | 6069 |
|  |  |  | 2 |  | 668 | 221 | 888 |  |
|  |  |  | 3 |  | 669 | 229 | 898 |  |
|  |  |  | 4 |  | 671 | 231 | 903 |  |
|  |  |  | 5 |  | 669 | 231 | 900 |  |
|  |  |  | 6 |  | 676 | 228 | 904 |  |
| shotgun |  |  | 1 | 668 | 83 | 199 | 283 | 2374 |
|  |  |  | 2 |  | 82 | 201 | 283 |  |
|  |  |  | 3 |  | 82 | 199 | 281 |  |
|  |  |  | 4 |  | 81 | 199 | 281 |  |
|  |  |  | 5 |  | 88 | 198 | 286 |  |
|  |  |  | 6 |  | 82 | 209 | 292 |  |
| SIP | Low | 10MM | 1 | 557 | 575 | 458 | 1033 | 6771 |
|  |  |  | 2 |  | 566 | 454 | 1019 |  |
|  |  |  | 3 |  | 568 | 474 | 1042 |  |
|  |  |  | 4 |  | 564 | 495 | 1059 |  |
|  |  |  | 5 |  | 566 | 485 | 1051 |  |
|  |  |  | 6 |  | 568 | 435 | 1003 |  |
| shotgun |  |  | 1 | 562 | 69 | 371 | 440 | 3239 |
|  |  |  | 2 |  | 69 | 376 | 445 |  |
|  |  |  | 3 |  | 68 | 357 | 425 |  |
|  |  |  | 4 |  | 69 | 391 | 459 |  |
|  |  |  | 5 |  | 68 | 405 | 473 |  |
|  |  |  | 6 |  | 68 | 365 | 433 |  |
| SIP | Medium |  | 1 | 642 | 642 | 584 | 1226 | 7695 |
|  |  |  | 2 |  | 648 | 475 | 1123 |  |
|  |  |  | 3 |  | 643 | 507 | 1150 |  |
|  |  |  | 4 |  | 652 | 571 | 1223 |  |
|  |  |  | 5 |  | 652 | 466 | 1119 |  |
|  |  |  | 6 |  | 641 | 566 | 1207 |  |
| shotgun |  |  | 1 | 651 | 81 | 411 | 492 | 3419 |
|  |  |  | 2 |  | 82 | 352 | 434 |  |
|  |  |  | 3 |  | 85 | 369 | 454 |  |
|  |  |  | 4 |  | 83 | 385 | 468 |  |
|  |  |  | 5 |  | 80 | 383 | 463 |  |
|  |  |  | 6 |  | 82 | 370 | 451 |  |
| SIP | High |  | 1 | 663 | 690 | 455 | 1145 | 7371 |
|  |  |  | 2 |  | 682 | 403 | 1084 |  |
|  |  |  | 3 |  | 686 | 408 | 1094 |  |
|  |  |  | 4 |  | 695 | 406 | 1101 |  |
|  |  |  | 5 |  | 684 | 432 | 1115 |  |
|  |  |  | 6 |  | 684 | 415 | 1099 |  |
| shotgun |  |  | 1 | 689 | 90 | 374 | 464 | 3487 |
|  |  |  | 2 |  | 90 | 373 | 462 |  |
|  |  |  | 3 |  | 92 | 370 | 462 |  |
|  |  |  | 4 |  | 89 | 361 | 450 |  |
|  |  |  | 5 |  | 87 | 361 | 447 |  |
|  |  |  | 6 |  | 89 | 388 | 477 |  |

^a^ Fragmentation of the reference genomes. Only performed once per simulation.

^b^ Calculation of abundance of each fragment within the BD window and writing the fragment abundance table to a file.

^c^ Simulation of Illumina reads and writing reads to a file in FASTA format.

**Table S4:** Summary statistics for recovery of reads, contigs, and MAGs for labeled genomes in initial simulations.

| **Metagenome**  **type** | **Comm.**  **G+C** | **Seq.**  **depth** | **Genomes aligned**  **to contigs**  **(≥ 50% recovered)** | **Genomes aligned**  **to contigs**  **(≥ 90% recovered)** | **Genomes**  **recovered**  **as MAGs** |
| --- | --- | --- | --- | --- | --- |
| SIP | Low | 5MM | 74 | 53 | 64 |
| shotgun |  |  | 66 | 36 | 32 |
| SIP | Medium |  | 66 | 48 | 56 |
| shotgun |  |  | 61 | 33 | 36 |
| SIP | High |  | 59 | 41 | 45 |
| shotgun |  |  | 57 | 30 | 38 |
| SIP | Low | 10MM | 93 | 75 | 89 |
| shotgun |  |  | 85 | 69 | 69 |
| SIP | Medium |  | 87 | 68 | 73 |
| shotgun |  |  | 79 | 63 | 67 |
| SIP | High |  | 80 | 59 | 67 |
| shotgun |  |  | 78 | 60 | 56 |

**Supplementary Figures**


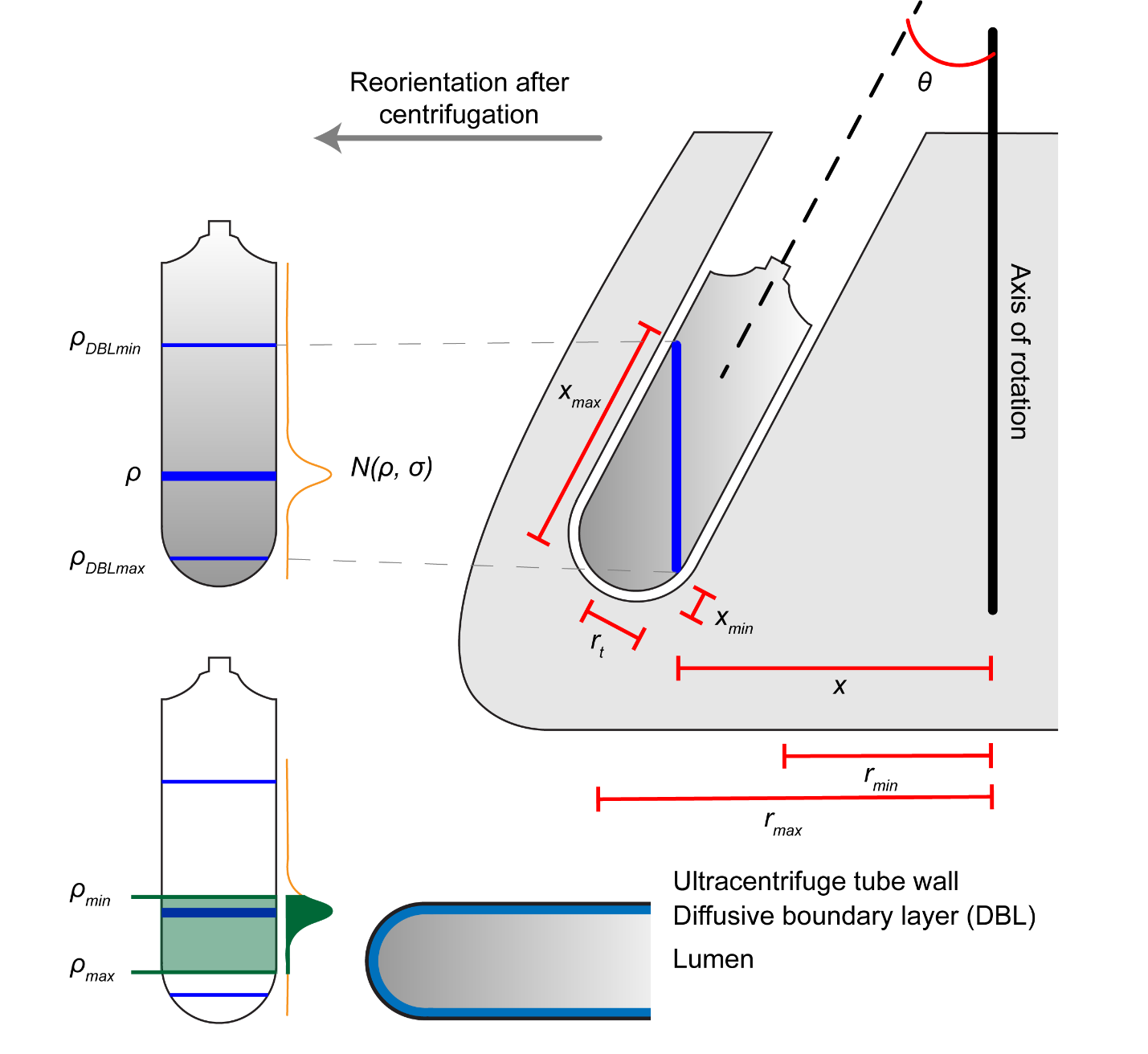


**Fig. S1:** Visual description of variables defined in the manuscript and table 2. Thick blue line indicates the position of a single DNA fragment during and after ultracentrifugation. Centrifugation setup shown here is based on a fixed angled rotor.

**Fig. S2:** Diagram of experimental design for case study simulations.


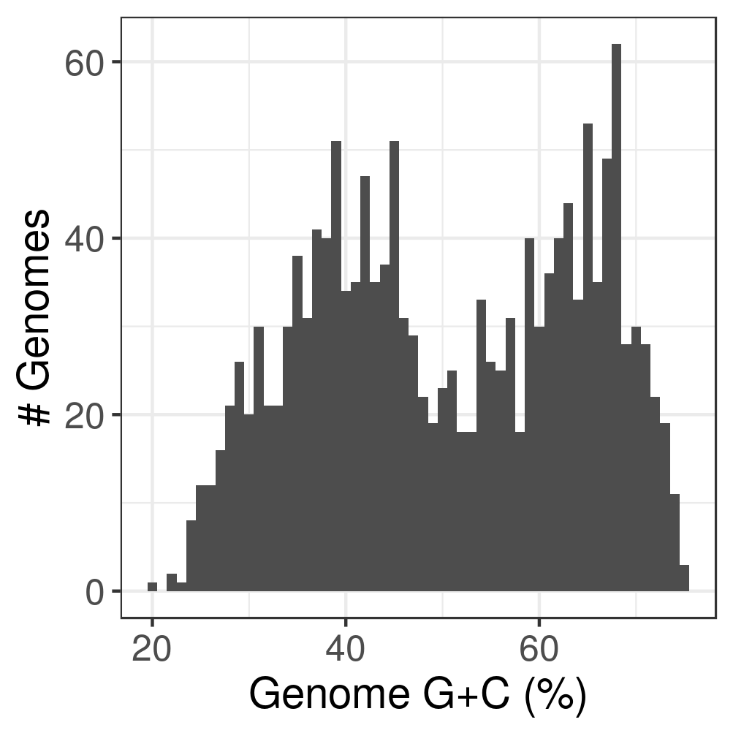


**Fig. S3:** RefSeq genomes G+C distribution with G+C bins rounded to nearest whole number (downloaded January 25, 2019).


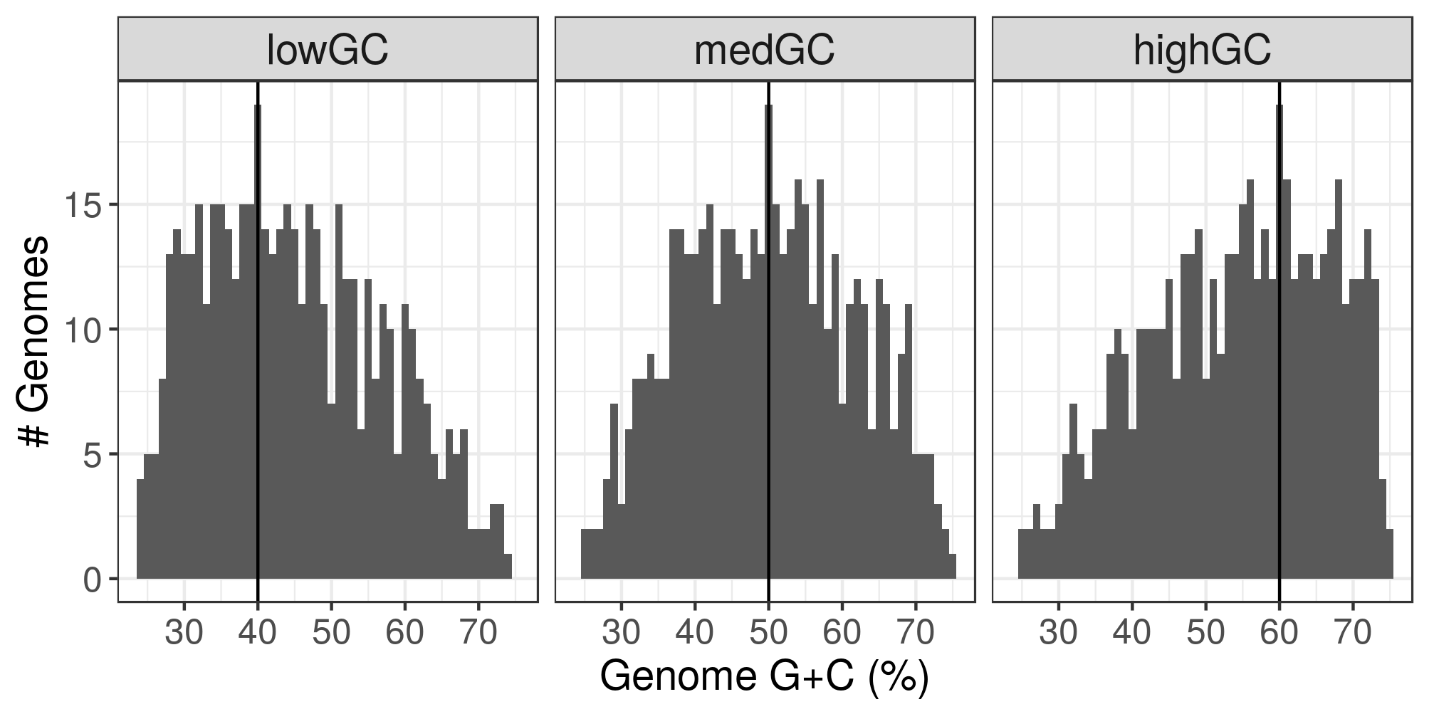


**Fig. S4:** G+C distributions for the 500 genomes in each reference set. G+C bins are rounded to the nearest whole number. Vertical line indicates G+C skew of the set.


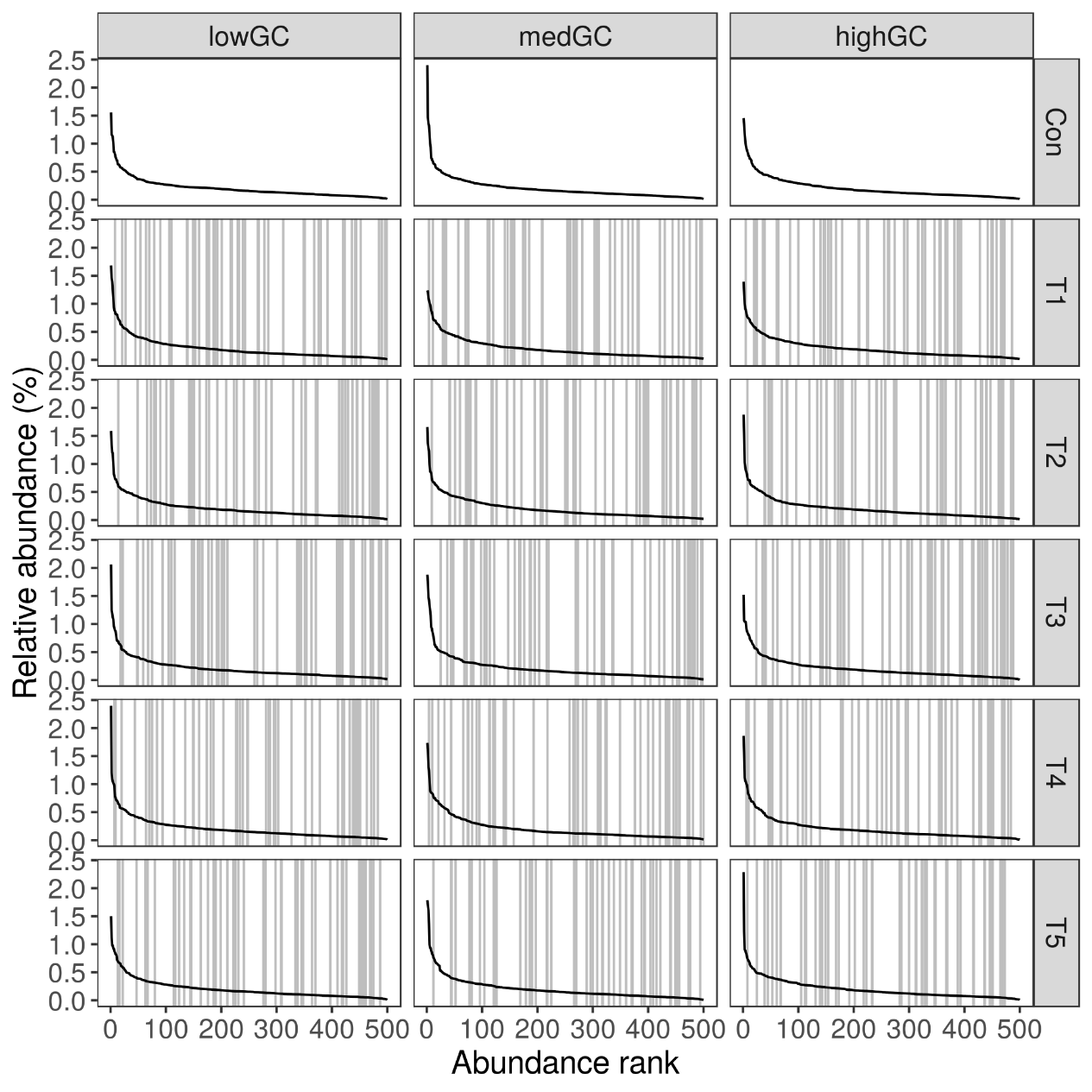


**Fig. S5:** Rank-abundance plots for each simulated sample or replicate community including the ^12^C-control (Con) and all five ^13^C-trials (T1-T5). Random genomes are only labeled in the ^13^C-labeled samples. Vertical grey lines indicate ranks of the 50 labeled genomes per treatment. Labeled genomes were selected such that mean ranks of the labeled genomes averaged across all samples were similar across the reference sets (lowGC, medGC, and highGC).


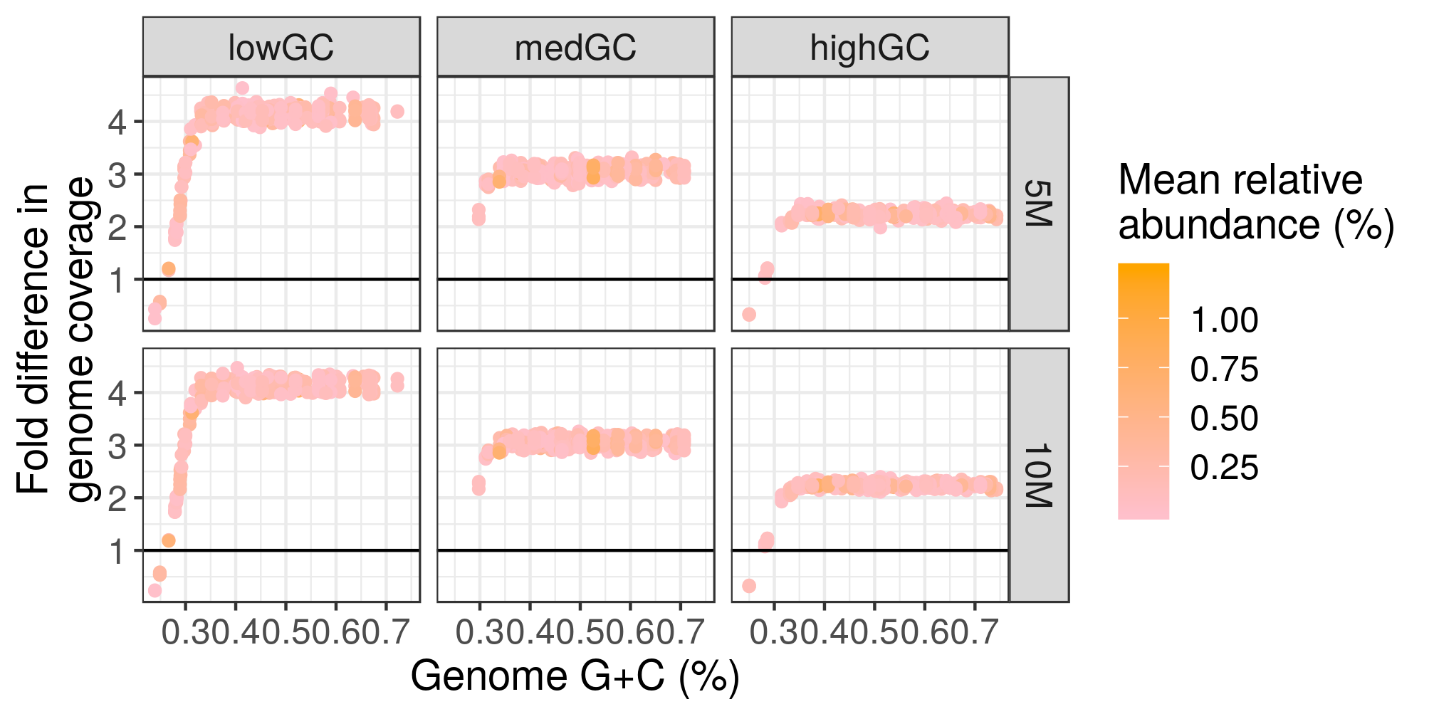


**Fig. S6:** Fold difference in raw read coverage for each labeled genome between the metagenomic-SIP and shotgun metagenomic libraries from the original simulations. Values above one indicate greater coverage in the metagenomic-SIP compared to the shotgun metagenomic libraries.


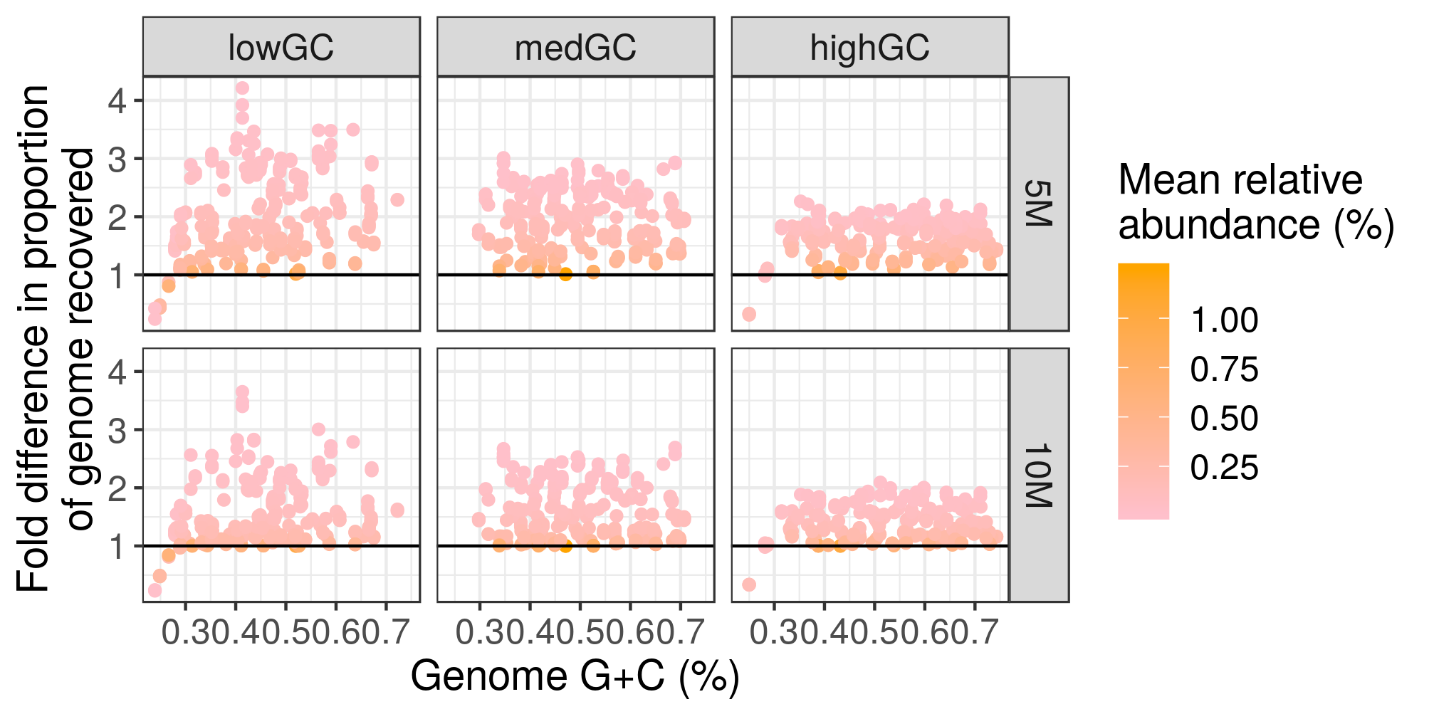


**Fig. S7:** Fold difference in proportion of each labeled genome recovered by reads between the metagenomic-SIP and shotgun metagenomic libraries from the original simulations. Values above one indicate greater recovery in the metagenomic-SIP compared to the shotgun metagenomic libraries.


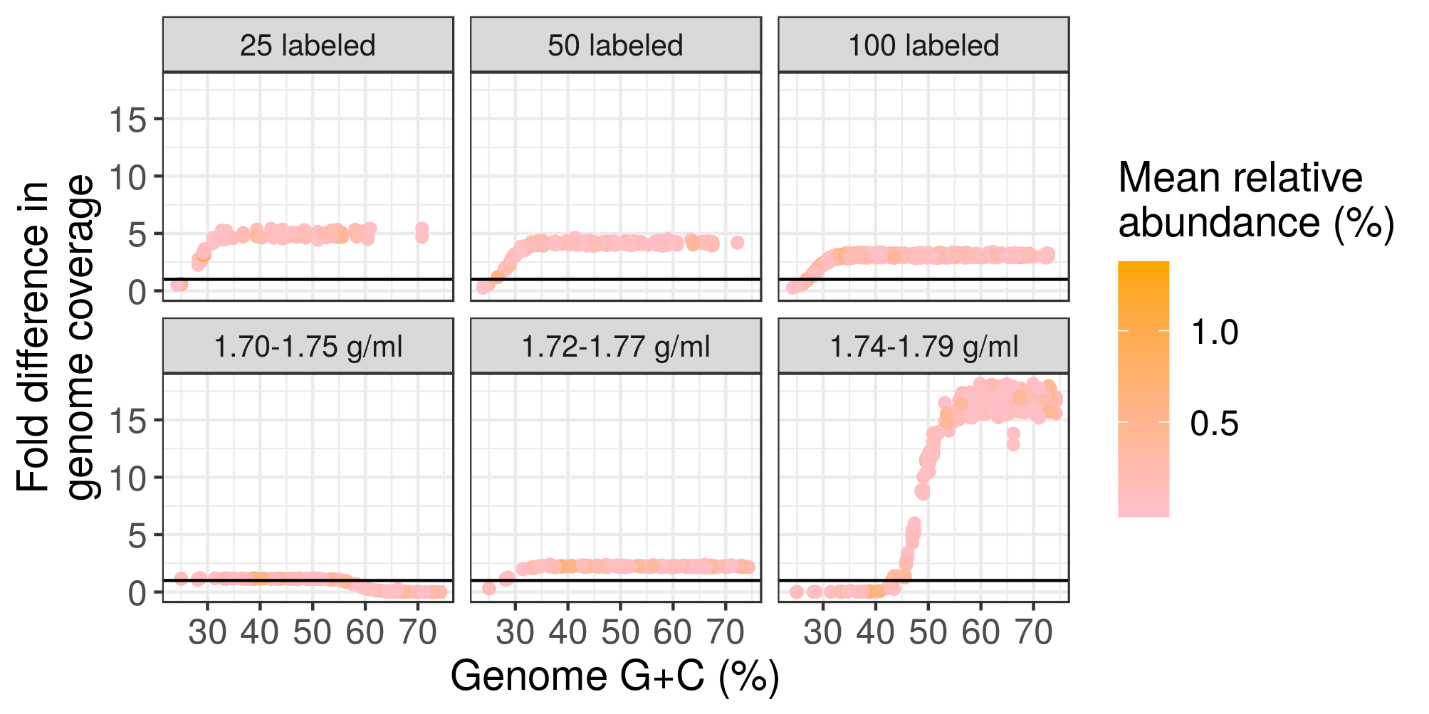


**Fig. S8:** Fold difference in raw read coverage for each labeled genome between the metagenomic-SIP and shotgun metagenomic libraries from the follow-up simulations. Values above one indicate greater coverage in the metagenomic-SIP compared to the shotgun metagenomic libraries. Simulation with the lowGC reference set with varying number of labeled genomes per sample is in the top row while the simulation with the highGC reference set with different sequencing window BD ranges is in the bottom row.


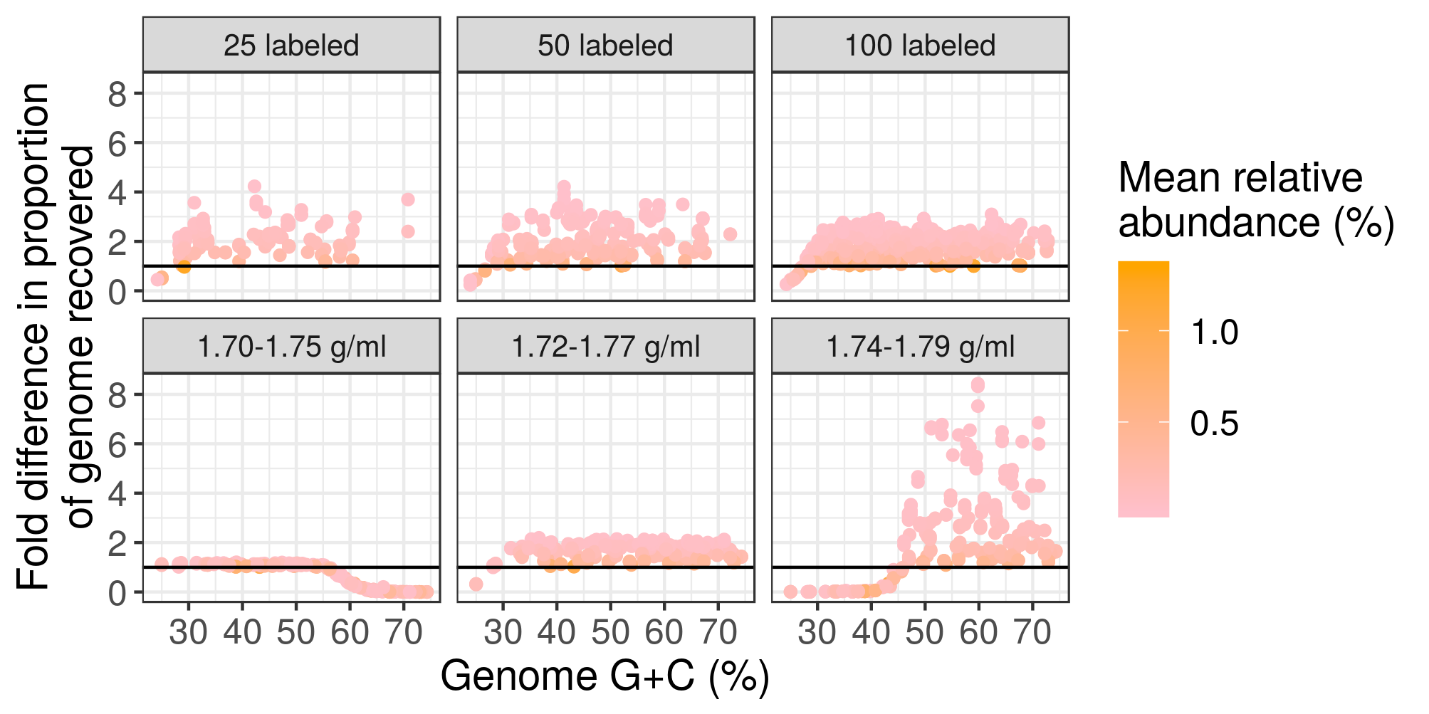


**Fig. S9:** Fold difference in proportion of each labeled genome recovered by reads between the metagenomic-SIP and shotgun metagenomic libraries from the follow-up simulations. Values above one indicate greater recovery in the metagenomic-SIP compared to the shotgun metagenomic libraries. Simulation with the lowGC reference set with varying number of labeled genomes per sample is in the top row while the simulation with the highGC reference set with different sequencing window BD ranges is in the bottom row.


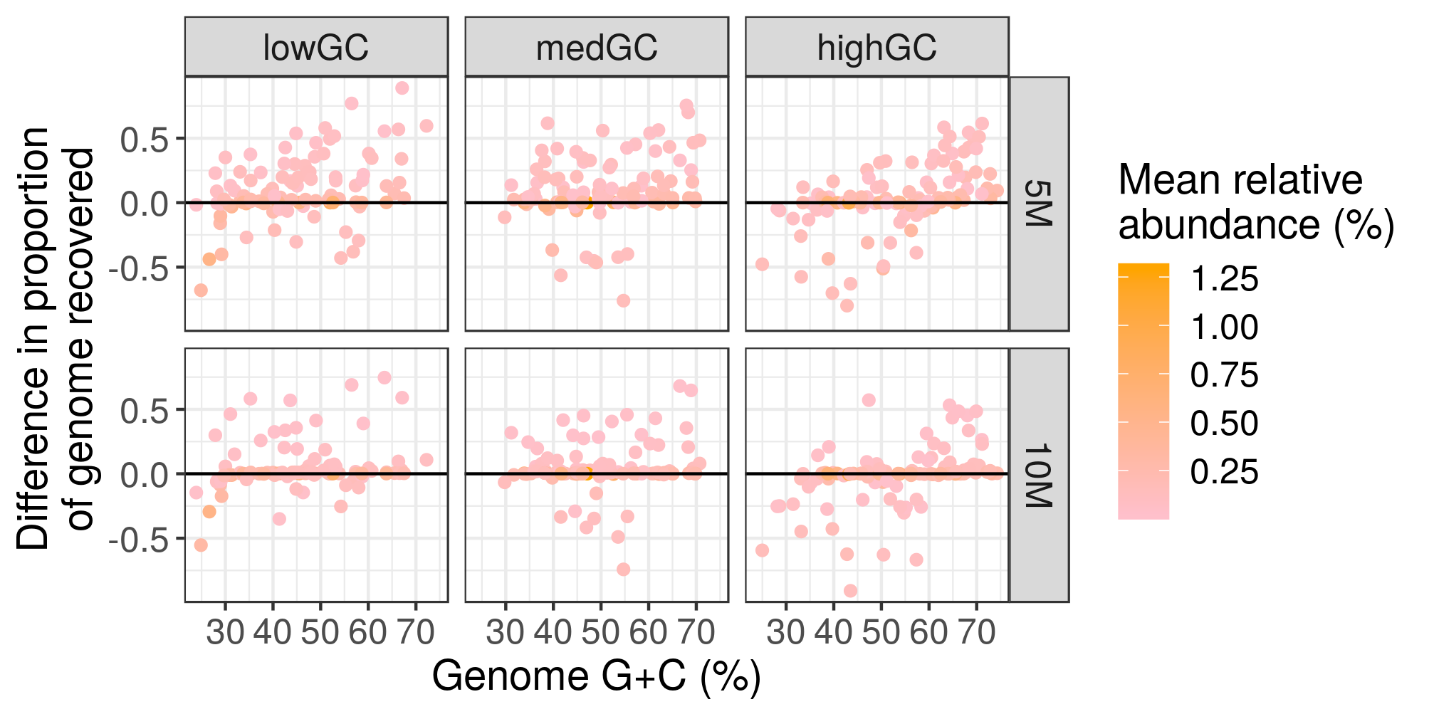


**Fig. S10:** Difference in proportion of each labeled genome recovered in co-assembled contigs between the metagenomic-SIP and shotgun metagenomic libraries from the original simulations. Values above zero indicate greater recovery in the metagenomic-SIP compared to the shotgun metagenomic contigs.


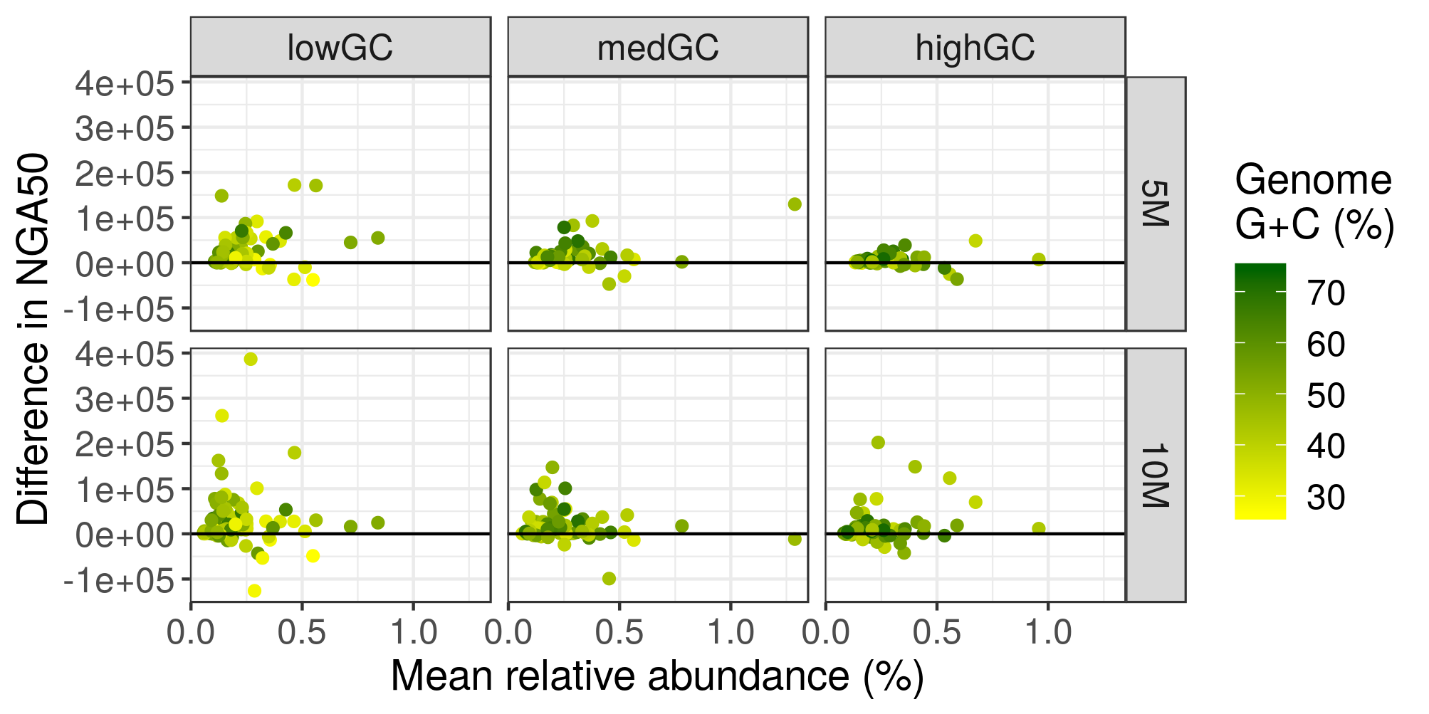


**Fig. S11:** Difference in NGA50 of each labeled genome covered by co-assembled contigs between the metagenomic-SIP and shotgun metagenomic libraries from the original simulations. Values above zero indicate greater NGA50 in the metagenomic-SIP compared to the shotgun metagenomic contigs. Only genomes with over 50% recovery in both SIP and shotgun metagenomes were used in this analysis.


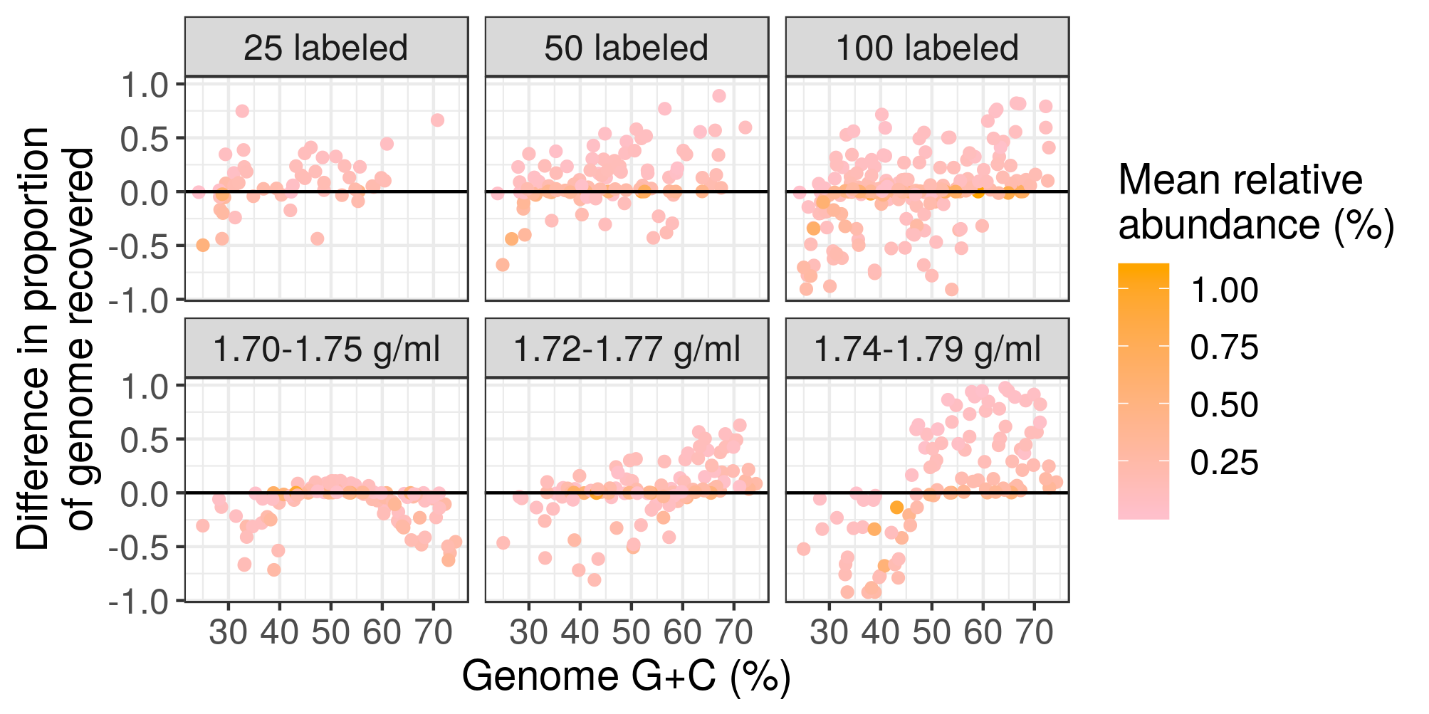


**Fig. S12:** Difference in proportion of each labeled genome recovered in co-assembled contigs between the metagenomic-SIP and shotgun metagenomic libraries from the follow-up simulations. Values above zero indicate greater recovery in the metagenomic-SIP compared to the shotgun metagenomic contigs. Simulation with the lowGC reference set with varying number of labeled genomes per sample is in the top row while the simulation with the highGC reference set with different sequencing window BD ranges is in the bottom row.


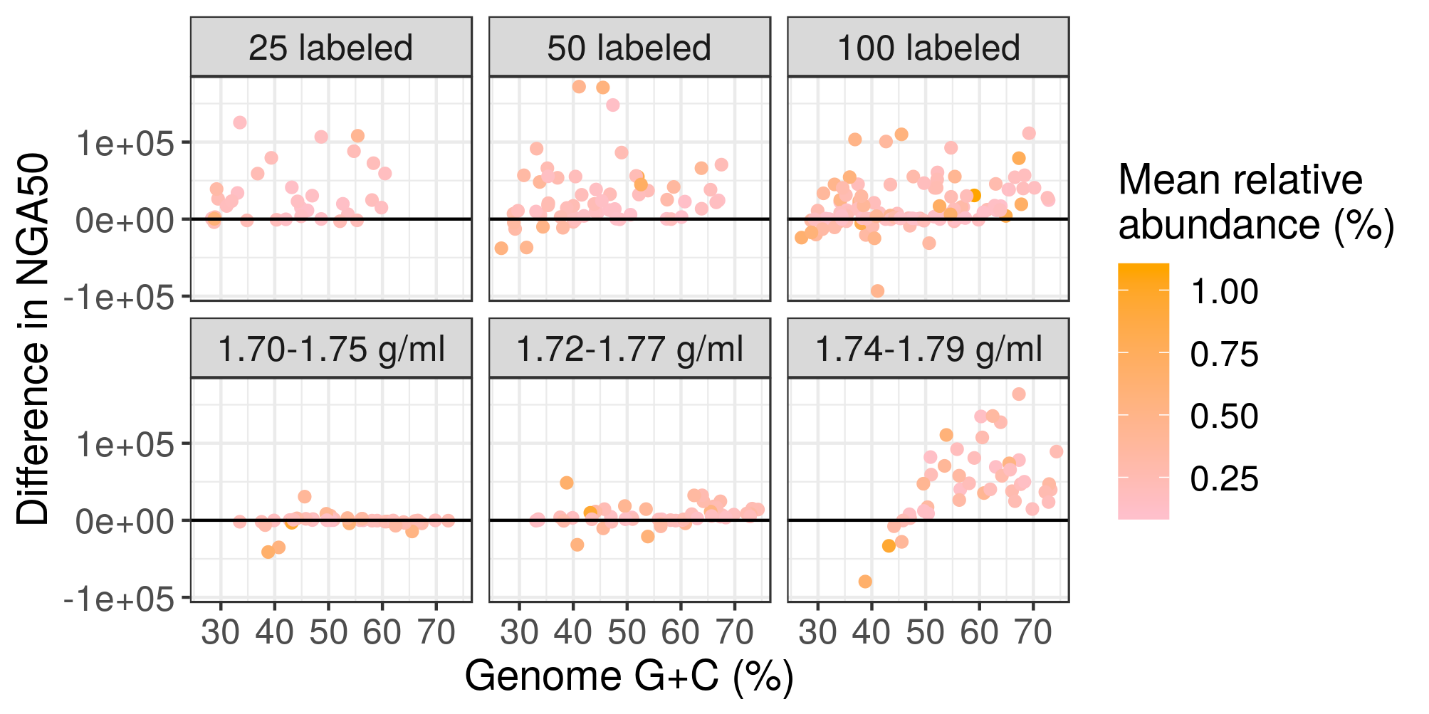


**Fig. S13:** Difference in NGA50 of each labeled genome covered by co-assembled contigs between the metagenomic-SIP and shotgun metagenomic libraries from the original simulations. Values above zero indicate greater NGA50 in the metagenomic-SIP compared to the shotgun metagenomic contigs. Only genomes with over 50% recovery in both SIP and shotgun metagenomes were used in this analysis. Simulation with the lowGC reference set with varying number of labeled genomes per sample is in the top row while the simulation with the highGC reference set with different sequencing window BD ranges is in the bottom row.


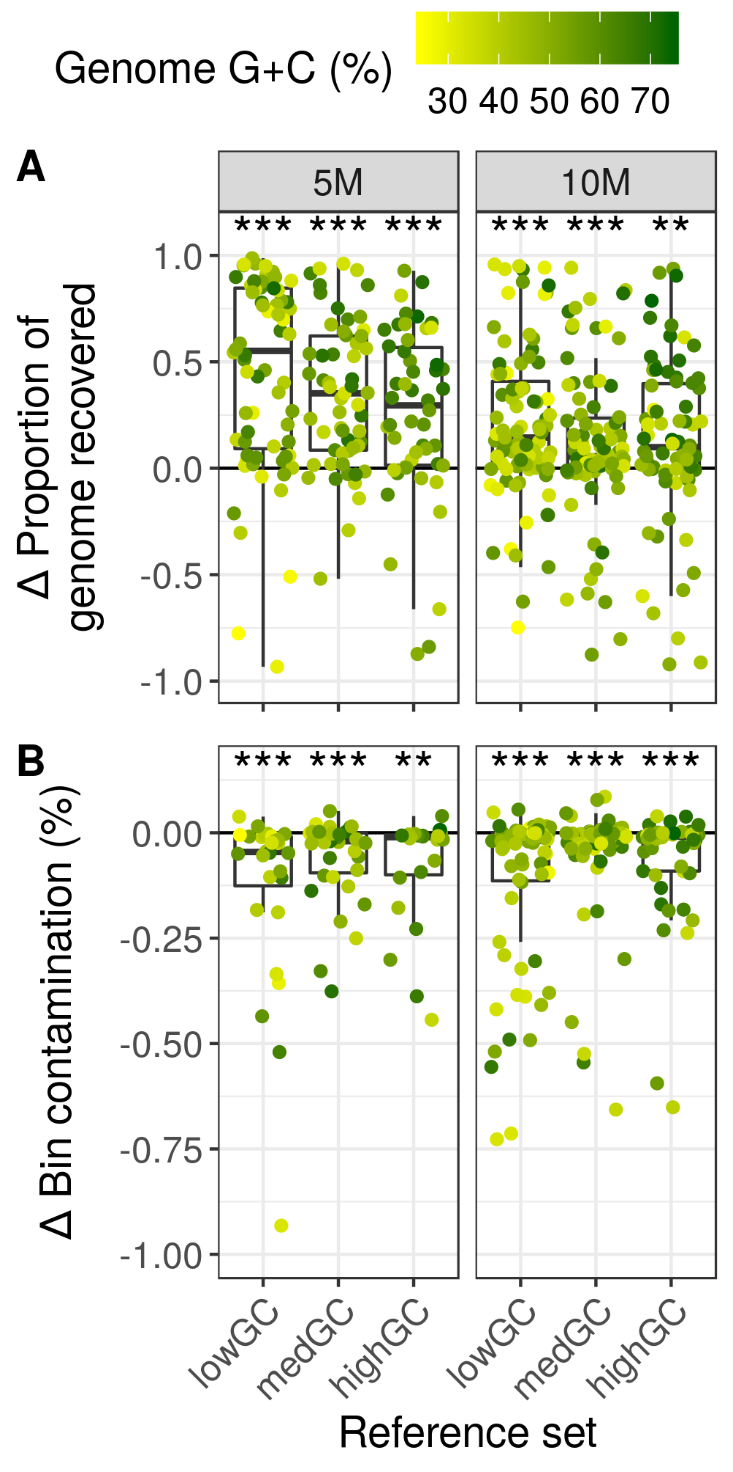


**Fig. S14:** Difference in binning quality between the SIP-metagenomes and shotgun metagenomes with the initial simulations using multiple bins per labeled genome. A) Difference in proportion of each labeled genome recovered in bins. B) Difference in the cumulative contamination for each labeled genome bin set.


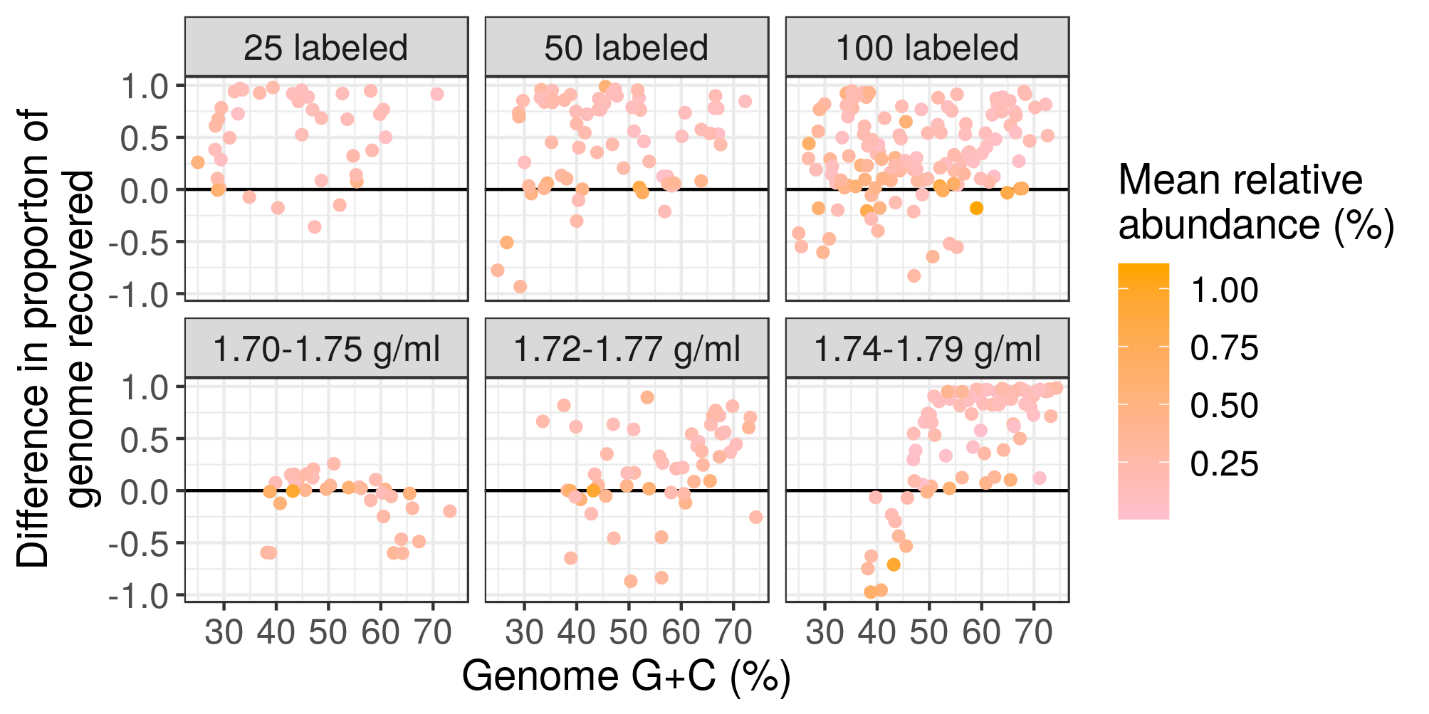


**Fig. S15:** Difference in proportion of each labeled genome recovered in a single most complete bin between the metagenomic-SIP and shotgun metagenomic libraries from the follow-up simulations. Values above zero indicate greater recovery in the metagenomic-SIP compared to the shotgun metagenomic bins. Simulation with the lowGC reference set with varying number of labeled genomes per sample is in the top row while the simulation with the highGC reference set with different sequencing window BD ranges is in the bottom row.
